# Clinical Reach into the Cognitive Space (CRITiCS): outline conceptual framework for safe use of generative artificial intelligence in mental health decision-making

**DOI:** 10.1192/bjb.2025.36

**Published:** 2026-04

**Authors:** Andrew Hider, Lesa Wright, Jacob Needle

**Affiliations:** 1 Iris Care Group, Cardiff, UK; 2 Psychiatry UK Ltd, Camelford, UK

**Keywords:** Artificial intelligence, case formulation, patient safety, computational psychiatry, clinical reasoning

## Abstract

**Aims and method:**

Advances in generative artificial intelligence, particularly through large language models, like GPT-4, have opened opportunities to develop cognitive agents to enhance clinical productivity, especially in complex secondary and tertiary care settings. However, as artificial intelligence begins to occupy the cognitive space traditionally held by human clinical reasoning, transparency becomes a significant concern. Unlike human decision-making, artificial intelligence-generated outputs may not be traceable to a transparent chain of clinical reasoning, potentially impacting safety if used without adequate ‘clinician reach’ into the reasoning space of artificial intelligence.

**Results:**

We highlight the need for a consensus framework to guide the responsible use of generative artificial intelligence in mental healthcare, which, it is argued, has cognitive demands and features distinct from physical medicine. We propose such a framework, Clinical Reach into the Cognitive Space (CRITiCS), to support clinician involvement in the deployment of these technologies.

**Clinical implications:**

This paper aims to spark dialogue and interest in both the clinical and artificial intelligence development communities.

‘… much of the meaning of a represented piece of information derives from the context in which the information is encoded and decoded. This can be a tremendous advantage. To the extent that the two thinking beings are sharing a common rich context, they may utilize terse signals to communicate complex thoughts.’ Douglas Lenat (1975)^1^

We will not review the entire history of artificial intelligence developments here, but it is clear that recent advances have surpassed initial timescale predictions as to the emergence of human-level cognitive processing, and perhaps even accelerated progress towards general artificial intelligence.^2^ For clinicians seeking a comprehensive overview that supports an understanding of how the new ‘transformer’-based large language models (LLMs) such as GPT4 work, we would suggest reading Wolfram^3^ as an adjunct to the ideas contained within this paper. By way of definition, a LLM is a type of algorithm capable of generating text based on a particular context due to the associated patterns in the vast quantities of data on which it has been trained. These patterns convey an apparent ability to reason when the LLM is presented with particular information.

In comparison with physical medicine, use cases for artificial intelligence in mental healthcare, while nascent, have been limited in scope given the differences between mental health treatment and those applied in physical medicine. For example, artificial intelligence use for diagnostic purposes in radiology is established, initial models have already been deployed and consensus governance frameworks developed.^4^ We would argue that this situation is due to both the ontological and procedural structure of mental healthcare, which we discuss below.

## Method

### Mental healthcare has unique features

It is important to delineate the distinct features of mental healthcare before considering how a conceptual model might support the development and governance of artificial intelligence systems that can be used safely in the clinical domain. It is also of note that the majority of conversations about the applications of artificial intelligence in mental health services have, to date, focused on clinical ‘expert models’ that replicate the (psychological) therapeutic task via chatbot interfaces. This paper starts from the position that ‘artificial intelligence therapy’ is not likely to be the main use case for generative artificial intelligence in complex mental health provision. Rather, we believe that the use case will centre on expert models that undertake “cognitive space” work such as case formulation, and therefore augment clinician cognition, to improve productivity and increase the time available for direct clinical contact. This is particularly relevant in secondary and tertiary care services, where information load and clinical administration burden are high, and where patient safety is a function of good global awareness of historical and current clinical factors. We also envisage that areas of the world with significant shortages of trained mental health clinicians would benefit from access to a generative artificial intelligence cognitive space that mirrors that of a highly trained clinician, particularly in the context of specialism-specific demand.

### Mental healthcare is ontologically and theoretically diverse

Mental healthcare delivery relies on the application of theories of mind and behaviour that are often contested and are pragmatic in nature, to a greater extent than those in physical medicine.^5^ Clinical constructs, derived from and constitutive of these theories, are used and manipulated in cognitive space by clinicians. These constructs do not typically map onto the world in the same way as those manipulated by clinicians working in physical medicine. For example, many, if not most, commonly used mental health ontological constructs such as, for example, ‘schemata’, ‘automatic thoughts’, ‘overvalued ideas’, ‘primary affect’ and ‘executive function’ are, we would argue, *heterotopic* to reality. In other words, these ontologies map reality onto often speculative or inferred psychophysical states, and/or onto higher-level factor analytically, neuropsychologically and/or biologically derived constructs from the fields of clinical psychology and psychiatry. Furthermore, constructs may be *ontologically distinct* across multiple theoretical areas (e.g. schemata is a construct used with variable meaning across different kinds of cognitive therapy and neuropsychological theory). It is therefore challenging to map a negative mental or emotional experience to a measurable causal chain. In contrast, physical medical constructs are often more measurable, visible and *homotopic* to reality (such as measures of neoplasm cell division in oncology). This makes it far simpler to map a negative experience such as a painful lump in the neck to a causal chain – for example, a neoplastic growth. We recognise that we are, to some extent, generalising and that, with respect to certain medical conditions (e.g. chronic inflammatory conditions such as fibromyalgia, and some chronic pain conditions), this is generally not the case. However, it is arguable that in mental healthcare, even quantitative psychometric and neuropsychometric data are not, at root, measuring ‘states of the world’ analogous to such observational measures in physical medicine. This feature of mental healthcare has profound implications for the safe clinical use of artificial intelligence. Given that clinical decision-making in mental healthcare uses, in almost all cases, these ‘heterotopic’ constructs, the transparency of a system’s ontology increases in importance, as does a governance framework that ensures that the model ‘knows’ the ontological status of the constructs it is representing, when constructing and mapping a high-dimensional cognitive space for the purposes of undertaking those cognitive tasks relevant to mental healthcare.

### Patient safety in mental healthcare is often a function of ‘weak signal’ detection in unstructured data-sets

Serious adverse events related to mental health, such as suicide and homicide, are rare in the general population. While the incidence of these is higher in specific clinical mental health populations, general purpose language models may not be equipped to identify these correctly in unstructured data-sets. The clinical record in mental health is dominated by unstructured data. ‘Weak signals’ in these data, such as a single sentence in a clinical note that reports a particular risk indicator, or patterns in collective clinical data across a single site, may in hindsight be seen as drivers of significant harm.^6^ Serious incident reviews in the field are replete with such examples.^7^ Arguably, AI-assisted automation of clinical history review is likely to improve signal detection. However, even given the known variabilities of humans in their ability to digest and detect weak signals in large data-sets, the accountability of the agent (the clinician) would still apply, post hoc (for example, in a serious incident investigation), even if a weak signal was missed by either an AI or a human agent. When applying artificial intelligence to mental health clinical review, a transparent governance framework is critical to ensure sufficient clinician ‘reach’ into the processes of signal *definition*, signal *detection* and the *activation of a response* to both weak and strong signals in the data, in order to maintain patient safety.^8^

Conversely, overemphasising weak signals due to lack of clinical experience can lead to iatrogenic harm through overtreatment and stigmatisation – for example, the misinterpretation of normative yet subjectively severe psychological distress and precipitous use of diagnosis and treatment. For example, this can occur in grief or adjustment disorder states, which may be misconstrued as severe depressive episodes. Any governance framework for the use of autonomous clinical agents will also need to be able to protect against this risk.

Large language models make it possible, depending on the interface used, for clinicians to question *in vivo* the information presented to them. This would permit trained clinicians to ask the right questions and properly examine weak signals. Furthermore, the domain knowledge that practising clinicians have would allow them to question the absence of signals where they might otherwise be expected. Taking this a step further, the clinician can explore the relationships between weak signals and related biopsychosocial factors, as established in the literature, with relative ease.

**Fig. 1:**
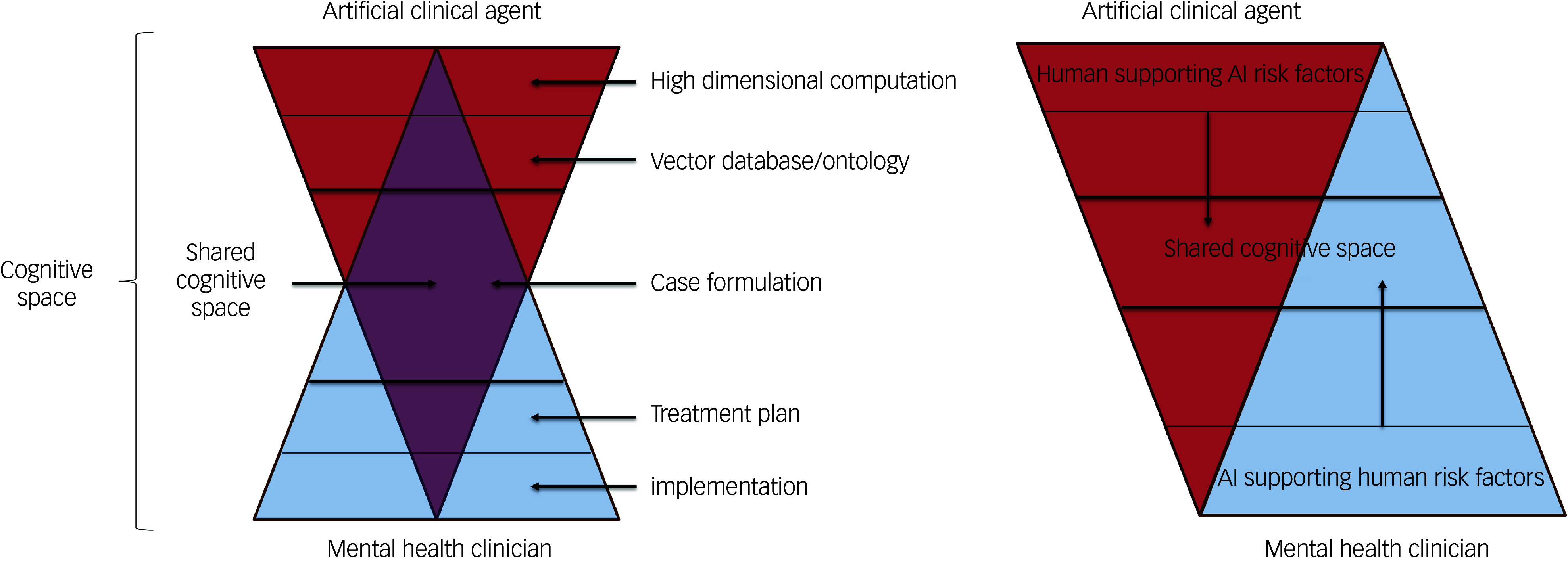
An outline proposal: the Clinical Reach into the Cognitive Space (CRITiCS) framework for generative artificial intelligence patient safety in mental healthcare. LLM, large language model.

### Mental healthcare delivery is driven by idiographic case formulation

There are some components of mental healthcare that yield to diagnostically based heuristics (for example, protocols for antipsychotic use in schizophrenia and other psychotic illnesses). However, in the majority of cases the overall treatment heuristic incorporates some degree of idiographic case formulation that drives ongoing treatment and risk management. Case formulation may either be theory naive – for example, generic ‘5P’ models as outlined by Winiarski;^9^ single-theory dependent – for example, cognitive behavioural formulation models;^10^ or procedurally theoretically integrated – for example, process-based therapy.^11^

The National Institute for Health and Care Excellence in the UK provides guidance on the broad theoretical constraints around which treatment for specific conditions should be provided. However, given that in mental healthcare, multiple morbidity is the norm, clinicians often undertake case formulation from a theory-diverse perspective in order to construct an individual map of the presenting difficulty, alongside the patient wherever possible. This supports treatment selection, clinical staging and outcome evaluation. Formulation is a highly idiographic task that depends heavily on clinician available knowledge, training level and both clinician and patient preference. There is limited predictive power to idiographic formulation, particularly in complex cases, such that a hindsight review can definitively conclude that the formulation was ‘wrong’ in a manner analogous to a treatment or assessment omission in the management of a physical disease. This has implications in the use of case formulations as ‘fine-tuning’ material for both clinicians and generative artificial agents. Further, as treatment histories elongate, multiple formulations typically exist in the clinical record.

Additionally, the passage of time may invalidate formulations, simply because they are missing more recent data points. Case formulations are substantially approximate representations of the clinical situation. Suitably skilled mental health clinicians have the ability to distil a sometimes vague sense of meaning into more concrete signals via learned clinical reasoning (for example, by noticing particular signs, inflections or case history details). This skill is hard to operationalise within the terms of a formal cognitive space in the context of mental healthcare, perhaps even more so than in physical healthcare. Finally, the nature of human mental health is that it is profoundly influenced by stochastic variables external to the person (such as social and economic circumstances and life events that may impact on the genesis and pathway of illness and, in many instances, define it). However, there have been recent advances in the literature supporting improved definition of this cognitive space.^12,13^

## Results

### The future – safe agentive reach into the clinical cognitive space

Computational models of the kinds of complex human cognition that account for all of the cognitive processes underpinning mental health clinical work remain matters of active investigation.^14^ Nonetheless, mapping and creating a high-dimensional computational space that broadly (albeit not fully transparently) models the cognitive space of a clinician when constructing a mental health case formulation is now technologically achievable, as is a system that automates the process of clinical formulation. However, there is a clear need to ensure that the ‘agentive reach’ of clinicians into the finalisation of hypothesis-driven case formulations constructed by artificially intelligent agents is significant and sufficient. There is also a need to ensure that objective and transparent confidence metrics are built into such processes, to reduce the risk of automation bias, i.e. clinician overconfidence in case formulations generated by such agents. Furthermore, future clinicians will need to be able to explain these metrics and their meaning to their patients. Development of confidence metrics and paradigms using current ‘explainable artificial intelligence’ protocols is an urgent task. Hauser et al,^15^ talking from the viewpoint of computational psychiatry, refer to this move towards increased process transparency as the development of ‘grey box’ models. Regulators and clinicians developing such tools may usefully develop objective measures of ‘reach’ using the proposed model as a general frame. The question of what is sufficient ‘clinician agentive reach’, and by what measure, into such automated clinical systems used in mental healthcare is an open one. We would suggest that all those seeking to develop new mental health clinical technologies based on LLMs should expect to be asked to demonstrate governance principles and measures across each of the domains of the framework (Fig. 1).

This diagram visualises an outline framework that could be used by practising clinicians without advanced knowledge of LLM and other artificial intelligence technology, to conceptualise the interaction between the traditional cognitive space of the mental health clinician and the new cognitive (clinical) space of the intelligent artificial agent.

The area space of each triangle represents the degree of cognitive space occupied by each agent (human and artificial intelligence). As such, it represents a combined human/machine cognitive space. As the area of cognitive space controlled by each agent *decreases*, the degree to which ‘supervisory’ attention is required by each agent (to the decision-making process) *increases*, to both support weak signal detection and ensure human penetrance into areas of case consideration that require, for example, empathic or derived relational responses originating in human (clinician) learning. Similarly, machine attention is deployed to some degree throughout the space, in order to support safety in areas where human cognitive and psychological weaknesses may affect the heuristic process.

In sum, solving the challenge of the ‘alignment problem’ as applied to artificial intelligence use in mental healthcare will involve both ensuring adequate human reach into machine cognitive spaces in healthcare applications of artificial intelligence, as well as using current technological advances to introduce intelligent agent ‘reach’ into the cognitive space of human clinical decision-making. This view sees artificial intelligence reach as a potential safety-enhancing process, given that human errors make such a large contribution to patient safety incidents in mental healthcare.

Table 1 provides an overview of the tasks and respective agentive reach of each agent in the range of cognitive spaces that would operate in artificial intelligence agent/human clinical tasks in mental healthcare.

**Table 1:** The Clinical Reach into the Cognitive Space (CRITiCS) model: nature and degree of agentive reach by cognitive space

Cognitive space	Artificial agent	Clinician role	Level of artificial intelligence agentive reach	Level of clinical agentive reach	Cognitive space risks (artificial intelligence)	Cognitive space risks (clinician)
LLM	Clinical reasoning using defined ontologies	Prior model training and supervision	High	Low	Hallucination of ontologies; human mentalisation ability absent, mentalisation not a part of the cognitive space; human supervision mitigates this	Automation bias; human intelligence insufficient to understand high-dimensional LLM cognition; human training to correctly use LLMs mitigates this
Vectorised database with contextual enrichment (e.g. Anthropic’s Contextual Retrieval)	Computation of high-dimensional data used to train model	Participation in system design; decision as to data requiring vectorisation; construction of ontology	High	Moderate	Opaque relationship between vectorised data and concept of ‘understanding’ clinical ontological constructs; need for real-time vectorisation of new data; application of metrics and explainable artificial intelligence techniques mitigates this	Insufficient attendance to data quality; biases and knowledge limitations may impact on construction of data-sets and ontologies
Clinical formulation process	Production of automated clinical formulation as product of LLM clinical reasoning on current and historic individual clinical data, using a trained artificial clinical agent	Clinical reasoning based on observation, assessment and review of clinical history	Moderate	Moderate/high	Outputs limited by model and ontology; weak signals may be missed if the clinician’s interrogation of the LLM output is poorly constructed due to lack of knowledge or inexperience	Human cognitive limitations impact on ability to conceptualise large quantities of data as complexity increases; high risk of missing weak signals; mitigated by training on weak signal principles
Treatment planning process	Suggestion of multidisciplinary treatment plan	Construction of agreed treatment plan; prescription of treatment plan	Low	High	Insufficient attendance to idiographic interpersonal factors derived from in-person interaction; potential mitigation with the patient clinician artificial intelligence triad^16^	Bias and impaired judgement; lack of awareness of totality of evidence base; high variability in clinical skill
Treatment implementation	Monitoring of treatment response via feedback from patient and clinician	Delivery of treatment	Low	High	Current technology cannot replicate emotional/interpersonal context of mental health treatment that is probably a significant factor in treatment response	High variability in clinical skill; self-serving bias; lack of attention to stop signals; lack of attention to iatrogenic harms

LLM, large language model.

## Discussion

The framework – that we have called ‘CRITiCS’ – suggests a potential conceptual basis through which to map existing artificial intelligence healthcare governance principles, such as those outlined by Reddy et al,^17^ to support the safe application of the new LLM technologies in the field of mental health. We have argued that mental healthcare needs such a framework, given its ontological and epistemological status as associated with, but distinct from, physical medicine. The clinical vision for such technologies must be a human one. As such, it is important that practising clinicians and users of mental health services – not just those with an understanding of technology – are involved in the development of the new computational tools. We believe that these tools will inevitably transform clinical practice in mental healthcare in the coming years. Clinicians and people receiving mental health services are, to paraphrase Lenat (1975), the ‘thinking beings’ most likely to ensure that this new artificial cognitive space is used safely and responsibly and in a manner that preserves the sound clinical reasoning, wisdom and interpersonal process that we would argue are the hallmarks of all good mental healthcare. We strongly advocate that users of mental health services must be involved as equal partners in the transformative changes yet to come. Psychological distress is still uniquely human. Most people either use, or love those who use, mental health services, so it is in all our interests for human partnerships to be the fulcrum of this process.

## Data Availability

Data availability is not applicable to this article as no new data were created or analysed in this study.
